# Activated interferon response from DNA damage in multiple myeloma cells contributes to the chemotherapeutic effects of anthracyclines

**DOI:** 10.3389/fonc.2024.1357996

**Published:** 2024-05-10

**Authors:** Jin Li, Zhuxia Jia, Rongxuan Wang, Bitao Xiao, Yanan Cai, Tianshu Zhu, Weiya Wang, Xinyue Zhang, Shu Fan, Xiaolong Fan, Wenmin Han, Xuzhang Lu

**Affiliations:** ^1^ Department of Hematology, Changzhou No. 2 People’s Hospital, The Affiliated Hospital of Nanjing Medical University, Changzhou, China; ^2^ Beijing Key Laboratory of Gene Resource and Molecular Development, Laboratory of Neuroscience and Brain Development, Beijing Normal University, Beijing, China

**Keywords:** multiple myeloma, DNA damage response, doxorubicin, drug resistance, type 1 interferon (IFN-I)

## Abstract

**Introduction:**

Multiple myeloma (MM) is a malignant plasma cell disease caused by abnormal proliferation of clonal plasma cells in bone marrow. Upfront identification of tumor subgroups with specific biological markers has the potential to improve biologically-driven therapy. Previously, we established a molecular classification by stratifying multiple myeloma into two subtypes with a different prognosis based on a gene module co-expressed with MCL-1 (MCL1-M).

**Methods:**

Gene Ontology (GO) analysis with differentially expressed genes was performed to identify signal pathway. Drug sensitivity was analyzed using the OncoPredict algorithm. Drug sensitivity of different myeloma cell lines was detected by CCK8 and flow cytometry. RNA-seq was performed on drug-sensitive cell lines before and after adriamycin treatment. RT-qPCR was used to further verify the sequencing results. The expression of γ-H2AX and dsDNA in sensitive and resistant cell lines was detected by immunofluorescence method.

**Results:**

In our study, we demonstrated that MCL1-M low MM were more sensitive to anthracyclines. We treated different myeloma cell lines with doxorubicin *in vitro* and discovered the association of drug sensitivity with IFN signaling. Herein, we demonstrate that the doxorubicin-sensitive myeloma cell line showed significant DNA damage and up-regulated expression of genes related to the IFN response, which was not observed in drug-insensitive cell lines.

**Discussion:**

Our results suggest that the active IFN signaling pathway may serve as a marker for predicting chemotherapy sensitivity in patients with myeloma. With our MCL1-M molecular classification system, we can screen patients with a potentially good response to the interferon signaling pathway and provide individualized treatment for MM. We propose IFN-a as adjuvant therapy for patients with myeloma sensitive to anthracyclines to further improve the therapeutic effect and prolong the survival of patients.

## 1 Introduction

Multiple myeloma (MM) is a malignant plasma cell disease caused by abnormal proliferation of clonal plasma cells in the bone marrow (1, 2). Individualization of myeloma management requires precise molecular classification, through which the risk of disease progression can be assessed independently of therapy, thus identifying whether patients are sensitive or resistant to treatment (3–5). However, how to accurately define dynamically evolving molecular subsets and implement stratified treatment of patients with different risk stratification remains a challenge.

The MCL1 gene co-expression module (MCL1-M) provides a possible solution to this problem. MCL1-M classifies MM patients into two different prognostic subgroups (MCL1-M low-risk MM and MCL1-M high-risk MM), and reflects different B cell differentiation and development pathways (6). MCL1-M low MM exhibits a gene expression profile that enriches the type I interferon (IFN) signaling pathway (6). Low expression of MCL1-M in patients with MM in anthracycline-based therapy had the same survival with or without bortezomib, suggesting that MCL1-M low MM are more sensitive to anthracyclines and reduce the survival advantage of bortezomib (6). However, the biological mechanism and clinical significance of IFN signaling enrichment in MCL1-M low MM are not fully characterized.

IFN-α has been used as a maintenance treatment for MM (7–9). Indeed, only small subsets of MM patients seem to benefit from this drug (10–12). Although the effects of IFN-α on myeloma are inconsistent, recent studies demonstrate that IFNs are critical in maintaining an effective antitumor response, and loss of IFN signaling results in resistance to treatment (13–15). When characterizing the effects of anthracycline-based chemotherapy, it was observed that type I IFN, as well as interferon stimulated genes (ISGs) were massively transactivated (16). Further research has shown that type I IFN is induced by a complex pathway that involves the release of nucleic acids from dying cells into the tumor environment (14, 16, 17).

To explore the relationship between IFN signaling and anthracycline therapy, we treated different myeloma cell lines with doxorubicin *in vitro* and observed the association between drug sensitivity and IFN signaling. Here, we found that the doxorubicin-sensitive myeloma cell line showed significant DNA damage and up-regulated expression of genes associated with the IFN response, which was not observed in drug-insensitive cell lines. Our results suggest that up-regulation of the IFN signaling pathway may be a marker to predict the sensitivity to chemotherapy in myeloma patients. We are able to select patients with a good response to the interferon signaling pathway through the molecular classification of MCL1-M, and provide individualized treatment for patients with low MCL1-M MM. We propose IFN-α as adjuvant therapy to patients with myeloma sensitive to anthracyclines to further improve the therapeutic effect and prolong the survival.

## 2 Results

### 2.1 MCL1-M low MM were enriched in type I interferon response genes and were more sensitive to anthracyclines

**Figure 1:**
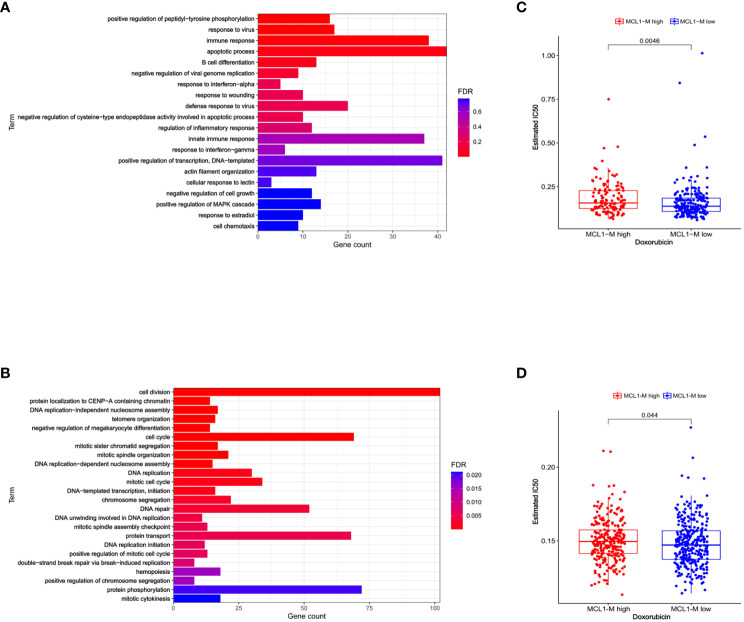
Biological process and drug sensitivity analysis among two risk groups. **(A)** GO analysis terms of MCL1-M low MM differentially expressed up-regulated genes. **(B)** GO analysis terms of MCL1-M low MM differentially expressed down-regulated genes. Data from GSE19784. **(C)** The box plots showing the estimated IC50 for doxorubicin between MCL1-M low and high MM. Data from the GSE19784 dataset. **(D)** The same analysis but data from GSE24080.

Previously, we established a molecular classification by dividing MM into two subtypes with distinct prognosis on the basis of a gene module co-expressed with MCL-1 (6). The survival analysis of all patients with anthracycline-based therapy suggested that the MCL1-M low group had a markedly better overall survival (OS) compared to the MCL1-M high group. We performed a Gene Ontology (GO) analysis with differentially expressed genes in the low and high MCL1-M groups and found that the upregulated genes in the low MM MCL1-M group were significantly enriched in immune-related biological processes, especially in the type I interferon response (**Figure 1A**), In contrast, the upregulated genes in the high MM MCL1-M were most significantly enriched in the cell cycle, DNA replication, and DNA repair (**Figure 1B**). Given that doxorubicin is a common treatment for MM (18–20), we wanted to assess the IC50 of this chemotherapy drug in two MCL1-M subtypes. We trained the predictive model on the data set from the GDSC cell line by ridge regression (21, 22). We estimated the IC50 of doxorubicin for each sample in GSE19784 and GSE24080 based on the predictive model. We observed a significant difference in doxorubicin sensitivity between low- and high-risk MCL1-M MM (**Figures 1C, D**). Obviously, low MCL1-M MM had significantly lower IC50 values for doxorubicin than those in high MCL1-M MM, indicating that patients in the low-risk MCL1-M group were more sensitive to doxorubicin. These results indicate that the MCL1-M is a unique genomic classifier that enables identifying the low-risk group with a unique biological process of activation of the interferon response, and thus predicting sensitivity to doxorubicin.

### 2.2 Doxorubicin-sensitive MM cell lines responded to interferon-α

**Figure 2:**
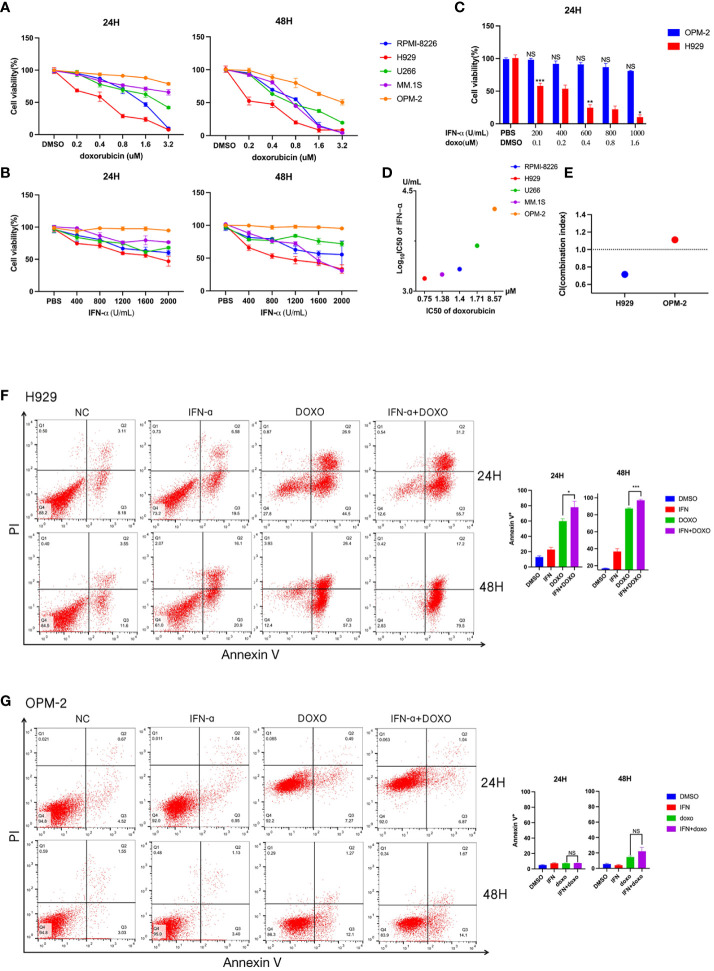
Detection of drug sensitivity of multiple myeloma cell lines. **(A)** Effects of doxorubicin on viability of different MM cell lines. Summarized cell viability assays are shown in MM cell lines treated with the indicated concentration for 24 or 48 hours. Results are normalized to cells treated with DMSO. **(B)** Effects of IFN-α on the viability of different MM cell lines. Five myeloma cell lines were treated with increasing concentrations of IFN-α for 24 or 48 hours. Results are normalized to cells treated with PBS. Each point in the graph represents mean ± SEM. **(C)** H929 and OPM-2 cell lines were treated with indicated concentration doxorubicin and IFN-α for 24 hours. **(D)** The horizontal axis represents the doxorubicin IC50 of five myeloma cell lines. The vertical axis represents these myeloma cell lines of IFN-α log_10_ IC50. **(E)** Combination index (CI) distribution between doxorubicin and IFN-α in H929 and OPM-2. **(F)** Apoptosis assays in H929 cells treated with doxorubicin and/or IFN-α for 24 or 48 hours. The concentration of IFN-α was 2000U/ml. The concentration of doxorubicin was 0.8μM. When the two drugs are combined, the concentration of IFN-α is 1000 U/ml and the concentration of doxorubicin is 0.4μM. Summarized data from three biological replications are shown on the right. **(G)** Apoptosis assays in OPM-2 cells treated with doxorubicin and/or IFN-α for 24 or 48 hours. The concentration was consistent with H929 cells. Summarized data from three biological replications are shown on the right. Experiments were repeated three times and representative results are shown. Error bars represent SEMs. *P <0.05, **P <0.005, ***P <0.0005, NS, no significance. NC, non-specific control.

To further explore the relationship between interferon response and doxorubicin sensitivity, we first assessed the effects of doxorubicin treatment on 5 MM cell lines for 24 or 48 hours. Subsequent cell viability assays revealed that different cell lines had different survival rates at the same concentration and treatment time (**Figure 2A**). According to the cell survival curve, the inhibitory effects of doxorubicin on myeloma cell lines were time-dependent and dose-dependent, in which H929 was drug-sensitive cell line and OPM-2 was drug-insensitive cell line. A previous study showed that anthracyclines stimulate malignant cells to rapidly produce type I IFNs rapidly (16). By binding to type I IFN receptors on neoplastic cells, type I IFNs trigger autocrine and paracrine circuits that result in the release of chemokines (16). Tumors lacking type I IFN receptors did not respond to doxorubicin (16). We then examined the proliferation of myeloma cells at different concentrations of IFN-α treated for different times. We found that IFN-α significantly increased the percentage of apoptosis in the five MM cell lines, in which H929 was a drug-sensitive cell line and OPM-2 was drug-insensitive cell line (**Figure 2B**). Together, these results showed that doxorubicin-sensitive MM cell lines responded to IFN-α. We combined the chemotherapy drug with IFN-α to treat sensitive and resistant cell lines (**Figure 2C**). Cell viability analysis revealed that the proliferation of drug-sensitive H929 cells was significantly inhibited by the combination with IFN-α, while the growth of drug-insensitive cells was not affected (**Figures 2D, E**). Subsequently, we treated myeloma cells with doxorubicin(0.8μM), IFN-α(2000U/ml), doxorubicin(0.4μM) + IFN-α(1000U/ml) for 24 hours and 48 hours, respectively. Our flow cytometric apoptosis assay showed that the combination of drugs with IFN-α significantly increased the rate of apoptosis in drug sensitive cells but did not affect apoptosis in drug-insensitive cells (**Figures 2F, G**), suggesting that doxorubicin-sensitive MM cell lines responded to IFN-α and combined therapy could further promote apoptosis of myeloma cells *in vitro*.

### 2.3 Doxorubicin activated the interferon response in drug-sensitive cell lines

**Figure 3:**
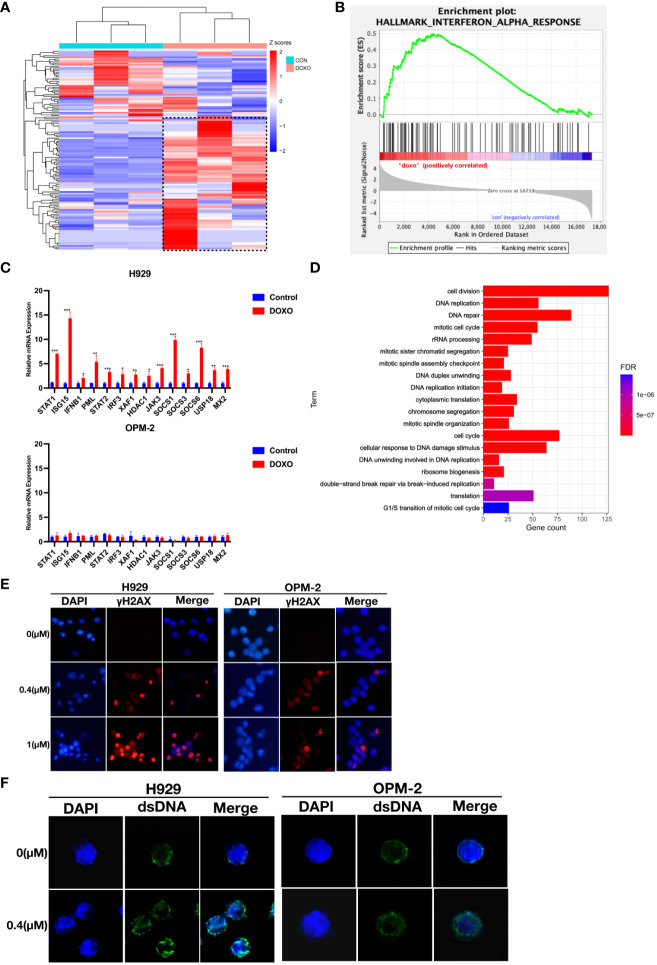
Doxorubicin specifically induces up-regulation of interferon response and is associated with dsDNA in the cytoplasm in response to DNA damage. **(A)** Heat maps for IFN-α response genes whose expression was altered in H929 by doxorubicin (0.4 μM, 48 hours). Means of three biological replications are shown. The gene names and expression have been included in the **Supplementary Table 1**. The color-scale represents the gene expression normalized by the Z-score. **(B)** GSEA of the genes involved in IFN-α response using the RNA-seq data from H929 treated with DMSO or doxorubicin. **(C)** qRT-PCR analysis of IFN-α response genes. Results are normalized to cells treated with DMSO. Means of three technical replications are displayed; error bars represent SEMs. *P <0.05, **P <0.005, ***P <0.0005. **(D)** GO functional enrichment analysis of down-regulated genes after doxorubicin treatment. **(E)** H929 and OPM-2 cells were treated with doxorubicin and then stained with DAPI and antibodies specific for γ-H2AX. **(F)** H929 and OPM-2 cells were treated with doxorubicin and then stained with DAPI and antibodies specific for dsDNA. Analyzes were duplicated, and representative immunofluorescence micrographs are shown. γH2AX appears as red. dsDNA appears as green. DNA appears as blue.

To elucidate the mechanism underlying the anti-myeloma effects of anthracyclines, we performed RNA sequencing (RNA-seq) using H929 cells from the doxorubicin sensitive cell line that were treated with doxorubicin (0.4 μM) for 48 hours. RNA-seq analysis showed that after doxorubicin treatment a series of genes associated with the interferon response were upregulated in H929 cells (**Figure 3A**). The relevant gene names and expressions have been included in the **Supplementary Table 1**.The significant impact of doxorubicin on IFN-regulated genes was further confirmed by GSEA (**Figure 3B**). These results suggest that type I IFN signaling may be associated with the anti-myeloma effect of doxorubicin. Using qRT-PCR, we confirmed that the expression of genes related to the interferon response was indeed up-regulated in H929 after doxorubicin treatment (**Figure 3C**). In contrast, these genes were not significantly upregulated in the drug-insensitive cell line OPM-2 after doxorubicin treatment (**Figure 3C**). These findings indicate that the type I IFN response in MM cells triggered by doxorubicin exerts an important antitumor effect and loss of this response can result in resistance to chemotherapy.

### 2.4 Disruption of DNA repair mediated chemoresistance of MM cells

Based on preclinical findings, the DNA damage repair process could trigger interferon-related immune responses (17, 23, 24). It is well known that anthracyclines could inevitably damage nuclear DNA (25–28). In our study, down-regulated differential expression genes in H929 after doxorubicin treatment were significantly enriched in DNA damage and DNA repair-related pathways (**Figure 3D**). This result indicated that DNA was seriously damaged and repair function was impaired in drug-sensitive cell lines. To investigate whether the degree of DNA damage was different in MM cells with different doxorubicin sensitivity, immunofluorescent staining of γ-H2AX was performed. Phospho-H2AX or γ-H2AX- is a marker of double-stranded DNA breaks (29, 30). Obviously, we observed higher fluorescence signals for γ-H2AX in H929 cells compared to the drug-insensitive cell line OPM-2 (**Figure 3E**), indicating that DNA damage was more severe in drug-sensitive cell lines, whereas drug-insensitive cell lines had strong DNA repair ability in the face of damage events. To confirm whether DNA damage caused by anthracyclines produces dsDNA (double-stranded DNA) that activates the interferon response in drug-sensitive cell lines. We stained myeloma cells with a dsDNA-specific antibody after anthracycline treatment and found that the accumulation of dsDNA in the cytoplasm was more evident in H929 cells than in OPM-2 cells (**Figure 3F**). These results confirmed that drug-insensitive cell lines have a better ability to repair damaged DNA in time, thus avoiding the interferon response.

## 3 Discussion

The major molecular mechanism of anthracyclines is their intercalation in DNA to inhibit topoisomerase II and cause DNA double strand breaks (DSBs) (31, 32). When cells encounter these DNA lesions, timely repair is needed to preserve function and survival (33, 34). Developing lymphocytes generate programmed DSBs at specific locations due to V(D)J recombination (35). This raises the question of whether there is a population of myeloma cells with a strong DNA repair ability that makes them insensitive to DNA damage drugs. Although the mechanism of action of anthracyclines has been characterized, drug resistance remains a challenge in effective anti-multiple myeloma therapy. We propose that inherently resistant cell lines, such as OPM-2, and patients in the MCL1-M high group may harbor intrinsic defects in the interferon signaling pathway. The OPM-2 cells exhibit prompt DNA repair mechanisms, facilitating continued cell proliferation despite the inflicted damage. The transient nature of DNA damage poses challenges in detecting interferon signals, as addition of IFN-α cannot impede the ongoing DNA repair process, resulting in the inability to reverse doxorubicin resistance. In our study, down-regulated differential expression genes in H929 after doxorubicin treatment were significantly enriched in DNA repair-related pathways. This result indicated that DNA was seriously damaged and repair function was impaired. Type I interferon signals are required for DNA damage response activation (35). Therefore, when we add exogenous IFN-α to severely damaged cell lines that further aggravate the DNA damage and lead to cell death. We found that the absence of an IFN response may be associated with drug resistance and provides potential combination treatment approaches in patients sensitive to doxorubicin.

The major conclusion of our study is that MCL1-M can identify a subpopulation of myeloma patients sensitive to anthracyclines, and *in vitro* results demonstrate that anthracycline-sensitive myeloma cells can be treated together with IFN-α therapy to further promote tumor cell apoptosis. Although previous studies have shown that anthracyclines can activate the IFN response, it requires an immunogenic death that relies on the immune system to trigger (16). In our study, anthracyclines induced the IFN response *in vitro* by releasing dsDNA into the cytoplasm after DNA damage. In addition, a previous study showed that sensitivity to DNA damage was coupled with sensitivity to IFNs such that selection for resistance to one leads to resistance to the other (36). To explore the differences in IFN response between resistant and sensitive cell lines, we focused on DNA damage repair. Analysis of transcriptomic changes in H929 cells after doxorubicin treatment identified a down-regulated DNA repair pathway. Recently, DNA repair has been reported to influence genomic changes and drug resistance in MM (33, 34, 37). Unrepaired DNA damage due to the aberrant DNA repair response in MM exacerbates genomic instability, enabling DNA release and drug resistance. Recent studies have found that amplification of 1q21 amplification improves the DNA repair ability of myeloma cells (38, 39). This finding is consistent with our findings in MCL1-M, that is, patients in MCL1-M were represented by 1q21 amplification. Furthermore, the expression of the DNA repair pathway in the MCL1-M high group was up-regulated compared to the MCL1-M low group. Unfortunately, we were unable to collect enough patient samples to validate our results.

In conclusion, the molecular classification of MCL1-M may allow us to identify the subgroups sensitive to anthracycline treatment, and the selection of IFN-α as adjuvant therapy in these patients further improves the therapeutic effect, reduces the dose of anthracyclines, and implements stratified treatment for patients with different subtypes of MCL1-M.

## 4 Materials and methods

### 4.1 Cell lines and reagents

Multiple myeloma cell lines MM.1S and OPM-2 were purchased from the Cell Bank (Chinese Academy of Sciences, Beijing, China). RPMI-8226, H929, and U-266 cells were kindly provided by Dr. Yang Yuan (Guizhou Provincial People’s Hospital). Cell lines were cultured in RPMI1640 medium (GIBCO; Thermo Fisher Scientific) supplemented with 10% FBS (GIBCO; Thermo Fisher Scientific), 100 U/mL of penicillin, and 100 mg/mL of streptomycin (Sigma-Aldrich). Doxorubicin was purchased from Selleckchem and resuspended in DMSO. Interferon-α (IFN-α, SRP4595, Sigma) was reconstituted in water. The Trizol RNA extraction reagent was purchased from Thermo Fisher Scientific. Anti-γ-H2AX (ab81299) was purchased from Abcam (Cambridge, UK). Anti-dsDNA (SC-58749) was purchased from Santa Cruz Biotechnology.

### 4.2 Drug treatment and cell viability assays

To evaluate the antiproliferative effects of doxorubicin and IFN-α, MM cell lines (5×10^3^ to 1×10^4^ cells/well in 96-well plate) were treated with a single drug or a combination of the two drugs or with DMSO for 24 or 48 hours. Cell viability was evaluated using a Cell Counting Kit-8 (Dojindo, Kumamoto, Japan) according to the manufacturer’s instructions. The CI value was evaluated by CompuSyn software.

### 4.3 Apoptosis assay

Apoptosis was evaluated by Annexin-V/PI staining and flow cytometric analysis using an ApoScreen Annexin V Apoptosis Kit (Southern Biotech, Birmingham, AL, USA) according to the manufacturer’s instructions. Data were analyzed using FlowJo software version 10.

### 4.4 Reverse transcription and quantitative real-time PCR

Total RNA from multiple myeloma cells was extracted with TRIzol (Thermo Fisher Scientific) following the manufacturer’s instructions. Subsequently, reverse transcription of 1 µg RNA into cDNA was carried out using a Hiscript II Q RT SuperMix reagent kit (Vazyme, China). The RT-qPCR assay was then performed with this mixture using SYBR Green PCR Master Mix (Roche Diagnostics, Basel, Switzerland) in a 96-well PCR plate (Nest Biotechnology, Wuxi, China).

### 4.5 Immunofluorescence assays

The cells were seeded in 6-well plates at a density of 2×10^5^ cells/well and cultured at 37°C for 24 hours. Doxorubicin at different concentrations was added to the 6-well plates for 24 hours. After treatment, cells were collected and incubated in poly-L-lysine-coated slides for 1 h at 37°C. The cells were then fixed in 4% paraformaldehyde for 20 min at room temperature, permeabilized with 0.5% Triton X-100 for 15 min and blocked with 1% BSA dissolved in PBS for 1 h. Cells were then incubated with anti-γ-H2AX or a primary antibody against dsDNA overnight at 4°C, followed by goat anti-mouse IgG conjugated secondary antibodies for 1 h at room temperature in the dark. The nuclei were stained with 0.1 μg/mL DAPI for 5 min. The cells were viewed using a fluorescent microscope.

### 4.6 RNA-seq analysis

H929 cells were cultured for 48 hours in the presence or absence of doxorubicin. RNA was extracted and used for library construction and then submitted to the Illumina Novaseq 6000 platform for sequencing. Differentially expressed genes (DEG) before and after drug treatment were identified using the DESeq2 package (version 1.36.0) (40). GO and Kyoto Encyclopedia of Genes and Genomes (KEGG) analyses were employed to distinguish the different biologic functions and pathways between DEGs. Gene set enrichment analysis was performed using the Molecular Signature Database (MSigDB) (41). The R package ‘OncoPredict’ of 402 drugs was used to predict *in vivo* drug responses in patients with high and low MCL1-M MM (21).

### 4.7 Statistical analyses

The statistical analysis was performed by using GraphPad Prism 9.0 (San Diego, CA, USA). The significance of differences was determined by two-tailed paired Student’s t-test. Data shown in the study were obtained from at least three independent experiments and probability values of p<0.05 were considered to be statistically significant.

## Data availability statement

The datasets presented in this study can be found in online repositories. The names of the repository/repositories and accession number(s) can be found in the article/**Supplementary Material**.

## Ethics statement

The studies involving humans were approved by The studies were approved by the Medical Ethics Committee of Changzhou No. 2 People’s Hospital. The studies were conducted in accordance with the local legislation and institutional requirements. The participants provided their written informed consent to participate in this study. Ethical approval was not required for the studies on animals in accordance with the local legislation and institutional requirements because only commercially available established cell lines were used.

## Author contributions

JL: Writing – original draft, Writing – review & editing, Conceptualization, Data curation, Formal analysis, Investigation, Methodology, Validation. ZJ: Data curation, Writing – original draft. RW: Data curation, Writing – original draft. BX: Data curation, Writing – review & editing. YC: Data curation, Writing – original draft. TZ: Data curation, Writing – review & editing. WW: Methodology, Writing – original draft. XZ: Methodology, Writing – original draft. SF: Software, Writing – original draft. XF: Writing – review & editing. WH: Project administration, Writing – review & editing. XL: Conceptualization, Funding acquisition, Investigation, Resources, Supervision, Visualization, Writing – review & editing, Writing – original draft.
